# Surgical management of primary hyperparathyroidism in Canada

**DOI:** 10.1186/s40463-014-0044-4

**Published:** 2014-11-01

**Authors:** Blair A Williams, Jonathan RB Trites, S Mark Taylor, Martin J Bullock, Robert D Hart

**Affiliations:** Division of Otolaryngology-Head & Neck Surgery, Department of Surgery, Dalhousie University, Halifax, NS Canada; Department of Pathology, Dalhousie University, Halifax, NS Canada

**Keywords:** Hyperparathyroidism, Parathyroidectomy, Multiple endocrine neoplasia

## Abstract

Primary hyperparathyroisim is a relatively common condition, for which the standard treatment is surgical excision of one or more of the parathyroid glands. Primary hyperparathyroidism can be due to a single adenoma or multiple gland hyperplasia. In recent decades localizing imaging has improved and there has been a shift away from multiple gland exploration toward a single gland excision. There are, however, no practice guidelines regarding an optimal approach to this condition. This study shows that there is a high degree of variation in practices across Canada and a large amount of uncertainty in the approach to primary hyperparathyroidism.

**Electronic supplementary material:**

The online version of this article (doi:10.1186/s40463-014-0044-4) contains supplementary material, which is available to authorized users.

## Findings

### Background

Primary hyperparathyroidism (PHPT) is an endocrine disease in which there is secretion of parathyroid hormone (PTH) outside the control of a physiological negative feedback loop. In the majority of patients the underlying cause is a parathyroid adenoma [1], while the remainder is due to multiple gland hyperplasia. Elevated PTH leads to elevated levels of calcium in the blood. The symptoms of PHPT are related, in part, to the elevated serum calcium. Surgical excision of the adenoma or hyperplastic glands generally cures the disorder.

Traditionally, the surgical approach to PHPT has been a bilateral exploration of the parathyroids and excision of abnormal glands. As imaging studies such as sestamibi scans have evolved and become more widely available, it has become feasible to preoperatively identify which of the four parathyroid glands is responsible for the PHPT. With this information, the surgeon could plan a limited dissection on one side of the neck and excise a single gland [2]. Greene et al. documented a shift in the preferred surgical approach to PHPT over a period of 10 years from 1998–2008, favouring a unilateral dissection at the end of the timeframe [3]. The use of rapid intraoperative PTH assays has also improved the accuracy of this approach, as the surgeon can detect a fall in PTH levels if the appropriate gland had been removed [4]. This technology is particularly useful in cases where preoperative imaging is unhelpful [5].

With changing technologies in preoperative assessment and intraoperative monitoring and a lack of evidence based guidelines regarding the management of PHPT, we hypothesized that there would be a high degree of variability in the management of this disease. We developed and distributed a survey to Otolaryngologists in Canada to document current practice patterns.

### Methods

Research ethics approval was obtained through the Capital District Health Authority Research Ethics Board. A survey was developed using Opinio software and encompassed three key areas: demographic data, preoperative investigations, and surgical approach. The questions from the survey can be found in the Additional file 1. The survey was circulated to local parathyroid surgeons and their feedback was incorporated to ensure internal validity. The final survey was circulated by email link to the membership of the Canadian Society of Otolaryngology – Head & Neck Surgery (CSO-HNS). A reminder email was sent out one month after the initial email. Responses were tabulated using the survey software.

### Results

The survey was distributed to 512 active members of the CSO-HNS and there were 68 respondents for a response rate of 13.3%. Of these respondents, 50% practiced in an academic setting, 37% in a community setting, and 13% had a mixed practice. In terms of fellowship training, 40% had no fellowship training, 34% had completed training in head and neck, 10% in endocrine surgery, and 16% had completed other fellowships. The geographic distribution of respondents approximately matched Canadian population distribution, with the majority of respondents practicing in Ontario (43%) and Quebec (22%).

**Figure 1:**
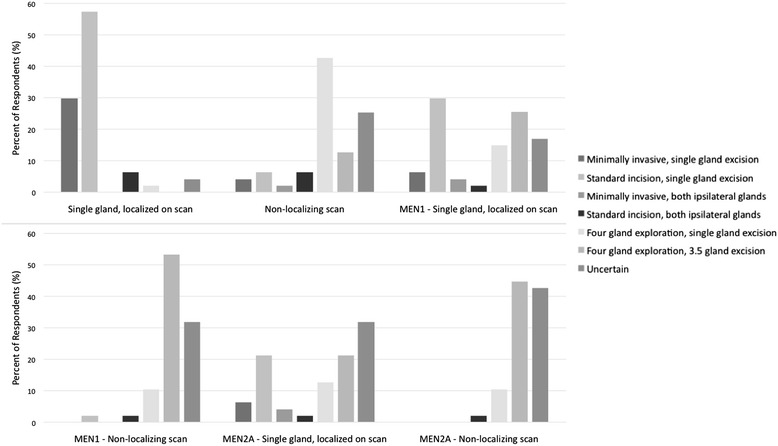
**Reponses to the preoperative investigation section of the survey.** Investigations are listed along the X-axis, with columns representing the frequency of use by respondents. PTH – parathyroid hormone, iCa – ionized calcium, CT – computed tomography, SPECT – single photon emission computed tomography.

Figure 1 summarizes the results of the portion of the survey regarding preoperative assessment. Preoperative bloodwork is fairly consistent but there is more variation in preoperative imaging. For intraoperative recurrent laryngeal nerve monitoring during parathyroidectomy, 45% of respondents never used it, 32% always used it and the remainder used the monitoring occasionally.

**Figure 2:**
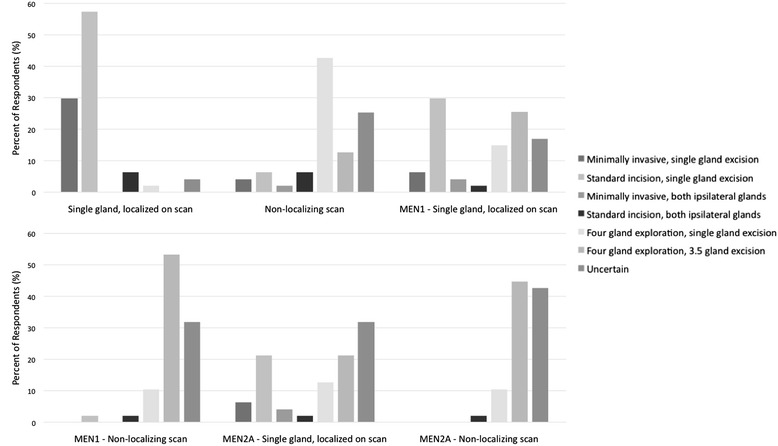
**Responses to the operative approach section of the survey.** Each scenario is listed along the X-axis, with the columns representing the percentage of respondents who chose each surgical management option listed in the legend. MEN – multiple endocrine neoplasm.

Figure 2 summarizes respondents approaches to 6 clinical scenarios: PHPT with and without a localizing scan, PHPT in multiple endocrine neoplasia type one (MEN1) with and without a localizing scan, and PHPT in MEN2A with and without a localizing scan.

### Discussion

The results of this survey illustrate that the approach to primary hyperparathyroidism is inconsistent. The response rate to the survey is deceptively low at 13.3%. The survey was distributed to all active members of CSO-HNS, the vast majority of whom do not perform parathyroid surgery. Though we could not access data on the total number of parathyroid surgeons in Canada, the number of respondents likely represents a significant proportion of Canadian parathyroid surgeons. Our respondents were a mix of academic and community based surgeons, as well as fellowship and non-fellowship trained surgeons. Regional distribution of responses by province roughly matched population patterns.

With respect to preoperative investigation for primary hyperparathyroidism (Figure 1), the initial bloodwork was consistent. There was considerable variability in the use of the various imaging technologies. This may be, in part, due to limited availability of highly specialized scans in non-academic centres. Likewise, there was a low rate of utilization of intraoperative PTH assays and intraoperative recurrent laryngeal nerve monitoring. This result contrasts to a 2002 survey, which found that 68% of respondents used intraoperative PTH assays [6]. The low rate of intraoperative PTH use may be due to surgeon preference or the resource may not be available at certain centres.

For the most common and straightforward scenario (single adenoma with a localizing scan) the surgical approach is consistent among respondents with 88% choosing a targeted excision through either a standard incision or a minimally invasive approach (Figure 2). This result is consistent with literature values, which demonstrate from 68% [3] to 92% [6] of surgeons choosing a limited approach in primary hyperparathyroid surgery.

For less common scenarios including non-localizing scans and primary hyperparathyroidism associated with MEN Types 1 and 2A, the surgical approach is inconsistent and there is a high degree of uncertainty among surgeons. The uncertainty seems to be in part related to surgical volume and fellowship training. The proportion of uncertain responses were higher among surgeons who performed less than 40 thyroidectomies per year and those respondents who had not completed either a head and neck or an endocrine fellowship. For example, in patients with a non-localizing scan, the uncertainty rate was 16.6% among fellowship trained surgeons and 29% among those without fellowship training. Among surgeons who did more than 40 thyroid surgeries per year the uncertainty rate was 20%, compared to 29% among lower volume surgeons. For patients with MEN2A and a non-localizing scan, the uncertainty rate was 33% among fellowship trained surgeons and 46% among those without fellowship training. Among surgeons who did more than 40 thyroid surgeries per year the uncertainty rate was 32%, compared to 52% among lower volume surgeons.

### Conclusion

This survey demonstrates significant variation in practice across Canada with respect to investigation of, and surgical approach to, primary hyperparathyroidism. There is also a high degree of uncertainty among surgeons as to the optimal approach to less common presentations. These results highlight the need for evidence based guidelines for primary hyperparathyroidism.

## Additional file

Additional file 1:
**Parathyroid surgery questionnaire.**

## Abbreviations

MEN: Multiple endocrine neoplasia
CSO-HNS: Canadian society of otolaryngology – head & neck surgery
PHPT: Primary hyperparathyroidism
PTH: Parathyroid hormone
