# Supplementary material for: Surgical management of primary hyperparathyroidism in Canada
Source: J Otolaryngol Head Neck Surg. 2014 Nov 1;43(1):44. doi: 10.1186/s40463-014-0044-4 (PMC4221664; doi:10.1186/s40463-014-0044-4)
Supplement: Additional file 1: — Parathyroid surgery questionnaire. [file 40463_2014_44_MOESM1_ESM.pdf]

Canadian practice patterns in parathyroid surgery

Demographics

Years in practice: ________

Type of practice: Academic/Community/Mixed

Province of practice: ________

Fellowship training: Head & Neck/Endocrine/Other/None

Number of thyroid surgeries per year:

0-5/5-10/10-20/20-40/40-60/80-100/>100

Number of parathyroid surgeries per year:

0-5/5-10/10-20/20-40/40-60/80-100/>100

Pre-operative Investigations

How often do you use the following in pre-operative planning:

PTH	Always/Occasionally/Never
Ionized calcium	Always/Occasionally/Never
Localizing scan	Always/Occasionally/Never
Bone scan	Always/Occasionally/Never
24h urine calcium	Always/Occasionally/Never
Serum creatinine	Always/Occasionally/Never
SPECT	Always/Occasionally/Never
Ultrasound	Always/Occasionally/Never
CT	Always/Occasionally/Never
MRI	Always/Occasionally/Never
Sestamibi	Always/Occasionally/Never

Intra-operative Approach

Do you use recurrent laryngeal nerve monitoring for parathyroid surgery? Yes/No

If yes, how often: 0-25%/25-50%/50-75%/75-100%

Do you use rapid intra-operative PTH assay? Always/Occasionally/Never

What is your surgical approach in the following scenarios (assume elevated PTH and elevated iCa^{2+}):

1. Single gland localizing on sestamibi:
   a. Minimally invasive single gland parathyroidectomy
   b. Standard incision single gland parathyroidectomy
   c. Minimally invasive resection of both ipsilateral parathyroids
   d. Standard incision resection of both ipsilateral parathyroids
   e. Four gland exploration with single gland parathyroidectomy
   f. 4 gland exploration with 3½ gland parathyroidectomy
   g. Uncertain

2. Non-localizing scan
   a. Minimally invasive single gland parathyroidectomy
   b. Standard incision single gland parathyroidectomy
   c. Minimally invasive resection of both ipsilateral parathyroids
   d. Standard incision resection of both ipsilateral parathyroids
   e. Four gland exploration with single gland parathyroidectomy
   f. 4 gland exploration with 3½ gland parathyroidectomy
   g. Uncertain

3. MEN1 with localizing scan positive for single gland
   a. Minimally invasive single gland parathyroidectomy
   b. Standard incision single gland parathyroidectomy
   c. Minimally invasive resection of both ipsilateral parathyroids
   d. Standard incision resection of both ipsilateral parathyroids
   e. Four gland exploration with single gland parathyroidectomy
   f. 4 gland exploration with 3½ gland parathyroidectomy
   g. Uncertain

4. MEN1 without positive localizing scan
   a. Minimally invasive single gland parathyroidectomy
   b. Standard incision single gland parathyroidectomy
   c. Minimally invasive resection of both ipsilateral parathyroids
   d. Standard incision resection of both ipsilateral parathyroids
   e. Four gland exploration with single gland parathyroidectomy
   f. 4 gland exploration with 3½ gland parathyroidectomy

    g. Uncertain

5. MEN2A with localizing scan positive for single gland
    a. Minimally invasive single gland parathyroidectomy
    b. Standard incision single gland parathyroidectomy
    c. Minimally invasive resection of both ipsilateral parathyroids
    d. Standard incision resection of both ipsilateral parathyroids
    e. Four gland exploration with single gland parathyroidectomy
    f. 4 gland exploration with 3½ gland parathyroidectomy
    g. Uncertain

6. MEN2A without positive localizing scan
    a. Minimally invasive single gland parathyroidectomy
    b. Standard incision single gland parathyroidectomy
    c. Minimally invasive resection of both ipsilateral parathyroids
    d. Standard incision resection of both ipsilateral parathyroids
    e. Four gland exploration with single gland parathyroidectomy
    f. 4 gland exploration with 3½ gland parathyroidectomy
    g. Uncertain
